# Rapid discrimination of pediatric brain tumors by mass spectrometry imaging

**DOI:** 10.1007/s11060-018-2978-2

**Published:** 2018-08-20

**Authors:** Amanda R. Clark, David Calligaris, Michael S. Regan, Daniel Pomeranz Krummel, Jeffrey N. Agar, Laura Kallay, Tobey MacDonald, Matthew Schniederjan, Sandro Santagata, Scott L. Pomeroy, Nathalie Y. R. Agar, Soma Sengupta

**Affiliations:** 1 Department of Neurosurgery, Brigham and Woman’s Hospital, Harvard Medical School, 60 Fenwood Road, 8016-J, Boston, MA 02115 USA; 2 Program in Chemical Biology, Harvard University, Cambridge, MA USA; 3 Therapeutics Graduate Program, Harvard Program in Therapeutic Science, Harvard Medical School, Boston, MA USA; 4 Winship Cancer Institute, Emory University School of Medicine, 1365C Clifton Road, Suite C5086, Atlanta, GA 30322 USA; 5 Department of Neurology, Emory University School of Medicine, Atlanta, GA USA; 6 Departments of Chemistry and Pharm. Sci., Barnett Institute, Northeastern University, Boston, MA USA; 7 Department of Pediatrics, Children’s Healthcare of Atlanta, Emory University School of Medicine, Atlanta, GA USA; 8 Department of Pathology and Laboratory Medicine, Children’s Healthcare of Atlanta, Emory University School of Medicine, Atlanta, GA USA; 9 Department of Pathology, Brigham and Woman’s Hospital, Harvard Medical School, Boston, MA USA; 10 Department of Neurology, Harvard Medical School, Boston Children’s Hospital, Boston, MA USA; 11 Department of Cancer Biology, Dana-Farber Cancer Institute, Harvard Medical School, Boston, MA USA; 12 Department of Hematology and Medical Oncology, Emory University School of Medicine, Atlanta, GA USA; 13 Department of Neurosurgery, Emory University School of Medicine, Atlanta, GA USA

**Keywords:** Medulloblastoma, Pineoblastoma, Mass spectrometry imaging, Biomarkers, Lipids, Brain tumors

## Abstract

**Purpose:**

Medulloblastoma, the most common primary pediatric malignant brain tumor, originates in the posterior fossa of the brain. Pineoblastoma, which originates within the pineal gland, is a rarer malignancy that also presents in the pediatric population. Medulloblastoma and pineoblastoma exhibit overlapping clinical features and have similar histopathological characteristics. Histopathological similarities confound rapid diagnoses of these two tumor types. We have conducted a pilot feasibility study analyzing the molecular profile of archived frozen human tumor specimens using mass spectrometry imaging (MSI) to identify potential biomarkers capable of classifying and distinguishing between medulloblastoma and pineoblastoma.

**Methods:**

We performed matrix-assisted laser desorption ionization Fourier transform ion cyclotron resonance mass spectrometry imaging on eight medulloblastoma biopsy specimens and three pineoblastoma biopsy specimens. Multivariate statistical analyses were performed on the MSI dataset to generate classifiers that distinguish the two tumor types. Lastly, the molecules that were discriminative of tumor type were queried against the Lipid Maps database and identified.

**Results:**

In this pilot study we show that medulloblastoma and pineoblastoma can be discriminated using molecular profiles determined by MSI. The highest-ranking discriminating classifiers of medulloblastoma and pineoblastoma were glycerophosphoglycerols and sphingolipids, respectively.

**Conclusion:**

We demonstrate proof-of-concept that medulloblastoma and pineoblastoma can be rapidly distinguished by using MSI lipid profiles. We identified biomarker candidates capable of distinguishing these two histopathologically similar tumor types. This work expands the current molecular knowledge of medulloblastoma and pineoblastoma by characterizing their lipidomic profiles, which may be useful for developing novel diagnostic, prognostic and therapeutic strategies.

**Electronic supplementary material:**

The online version of this article (10.1007/s11060-018-2978-2) contains supplementary material, which is available to authorized users.

## Introduction

Medulloblastoma (MB) is the most common primary pediatric malignant brain tumor, originates in the posterior fossa, and has high-grade embryonal features [1, 2]. Based on DNA aberrations and molecular profiling, MB tumors are subgrouped as either: Wnt-signaling pathway (WNT)-activated; Shh-signaling pathway (SHH)-activated, further subdivided into wild-type or mutant TP53; group 3; group 4 [3, 4]. Interestingly, there have been occasional reports whereby MB tumors may be misclassified as pineoblastoma (PB) when diagnosed based on histopathology without molecular or radiological information [5]. PB are rare malignancies that occur in the pineal gland [6]. Like MB, these tumors tend to occur in pediatric patients and display high-grade embryonal morphology [6]. PB has a propensity to spread through the cerebrospinal fluid and is associated with a poor prognosis [6].

Despite MB subgrouping, therapies for both MB and PB rely on maximal surgical resection with adjuvant radiotherapy and/or conventional cytotoxic chemotherapy [7, 8]. With the push to develop targeted and personalized therapeutics, a central focus is the discovery of biomarkers for improving clinical diagnoses, prognostic characterization, and prediction of therapeutic response [9, 10].

Abnormal lipid metabolism is a characteristic of cancer cells [11–13], and the potential to harness lipids for disease biomarkers has been investigated [14–16]. The differential abundance of various lipids is closely linked to key biological processes such as cellular signaling, cell membranes assembly, cell–cell interactions, and chemical-energy storage [17]. Advantages of using lipids as biomarkers include the well-established role of lipids in disease pathogenesis, including cancer [18–20]; stability of lipids when samples are archived at − 80 °C, thus allowing large scale studies of multiple patients [21]; and existence of well-established methods for lipid analysis by mass spectrometry (MS) and mass spectrometry imaging (MSI) [22].

Mass spectrometry techniques, like matrix-assisted laser desorption/ionization (MALDI), are utilized for lipid analyses. Molecules, including lipids, can be spatially resolved by MALDI MSI, which yields mass spectra in respect to specific coordinates of a sample to provide high spatial resolution maps of the distribution and relative intensity of molecules in tissue samples and allows comparison with conventional histology images. These comparisons can inform the molecular characterization of different components of tumors, including the cancer cells and the tumor associated stromal elements [23–25]. Moreover, the identity of lipid molecules are discernable using high-resolution mass analyzers, such as Fourier transform ion cyclotron resonance (FT-ICR) mass analyzers, which are capable of determining *mass-to-charge* (*m*/*z*) ratios with a mass accuracy below 1 part-per-million. An advantage of analyzing molecules with such high mass accuracy is the ability to infer the elemental composition of unknown species with high confidence. Using the proposed elemental composition, structural elucidation of isobaric species can be achieved through the interpretation of fragmentation patterns provided by tandem mass spectrometry (MS/MS) methods.

We have analyzed the metabolic profiles of high-grade embryonal tumors by high mass resolving MALDI 9.4T FT ICR MSI and applied multivariate statistical analyses, principal component (PCA) and receiver operating characteristic (ROC), to generate a list of candidate biomarkers with classifying power for MB and PB. We aimed to identify potential biomarkers for rapid diagnostic applications for distinguishing MB and PB. Furthermore, the comprehensive lipidomic analysis of MB and PB generated in this pilot study may yield insights into disease pathogenesis, and may provide lipid-based signatures for patient specific treatment.

## Materials and methods

### Samples

Human tissue samples (eight MB and three PB) were obtained surgically at time of initial diagnosis and before treatment, under IRB-approved protocols with informed consent. Samples were snap frozen and stored at − 80 °C. MB samples had previously been sub-classified using expression profiling data [26].

### Tissue preparation and staining

Tissue sections were prepared using a Microm HM550 cryostat (Thermo Fisher Scientific, Kalamazoo, MI) with microtome chamber and specimen holder at − 20 °C. Twelve-micron thick sections were mounted onto ITO-coated microscopic slides (Bruker Daltonics, Billerica, MA) for MALDI MSI or standard glass slides for hematoxylin and eosin (H&E) staining and allowed to dry 10 min in a desiccator.

### Histopathology evaluation

H&E stained sections were imaged at ×40 magnification using a Zeiss Z1 Observer microscope (Zeiss, Oberkochen, Germany) operating with Zen Pro Software (Zeiss, Oberkochen, Germany). Histopathological features in H&E stained sections were characterized by a board-certified neuropathologist.

### MALDI MS imaging

#### Matrix deposition

2,5-Dihydroxy benzoic acid (2,5-DHB, 160 mg/mL solution in methanol/0.1% TFA 70:30 vol/vol) or 1,5-diaminonaphtalene (1,5-DAN, 20 mg/mL solution in acetonitrile/H_2_O 70:30 vol/vol) was deposited using a HTX TM-Sprayer (HTX Technologies, Chapel Hill, NC): flow rate, 90 µL/min; spray nozzle velocity, 1200 mm/min; spray nozzle temperature, 75 °C; nitrogen gas pressure, 10 psi; track spacing, 2 mm; number of passes, 4. 2,5-DHB, 1,5-DAN, and TFA were obtained from Sigma-Aldrich (St. Louis, MO). Methanol, H_2_O, and acetonitrile were obtained from Fisher Scientific (Waltham, MA).

#### Mass spectrometry

Mass spectra were acquired using a 9.4T SolariX Fourier transform mass spectrometer (Bruker Daltonics, Billerica, MA). Images were acquired with a pixel step size for the surface raster set to 150 µm in FlexImaging 4.0 (Bruker Daltonics, Billerica, MA). Using serially sectioned tissue samples one image was acquired in positive ion mode and a second image acquired in negative ion mode. Laser power was set to 20% for positive mode and 15% for negative mode. Acquisition range was set to *m*/*z* 100–3000, 10 positions were sampled (25 shots/position at a laser frequency of 1000 Hz) within a given pixel for a total of 250 laser shots per pixel.

#### Imaging data processing and analysis

MALDI MS were analyzed using SCiLS Lab 2016b and 2018 Pro software (Bruker, Germany). Data preprocessing steps were performed as follows: mass spectra normalized to total ion count (TIC), an *m*/*z* interval width of 0.001 Da was selected, and weak denoising was applied. The segmentation pipeline was performed and resulted in an aligned peak list containing 143 *m*/*z* intervals. Multivariate analyses, including principle component (PCA) and receiver operating characteristic (ROC), were performed using the *m*/*z* intervals generated from the aligned peak list. Note: no scaling was performed for PCA. Ions from the PCA loadings plot were shown as single ion images. In ROC, random spectra subsets from MB and PB were used to define each tumor class. The resultant ions (*m*/*z* measured values) with an area under the curve (AUC) value above 0.75 were considered as MB classifiers, while AUC values < 0.25 were considered as PB classifiers. Ions determined from ROC analysis were searched in Lipid Maps [27] to provide tentative peak assignments. For ions with more than one potential assignment, assignment with lowest Δppm was chosen. Tentative peak assignments were made for ions with a Δppm < 2.0. ROC ions that met this criterion were visualized as single ion images. The total time used for the high spectral resolution mass spectrometry imaging experiments and multivariate data analyses performed herein was approximately 72 h. However, a more rapid approach could be translated from these findings in which the time from sample acquisition (biopsy) to diagnosis (characterization by MALDI MSI) could be achieved in less than ten minutes.

## Results

### Discrimination of medulloblastoma and pineoblastoma

**Fig. 1:**
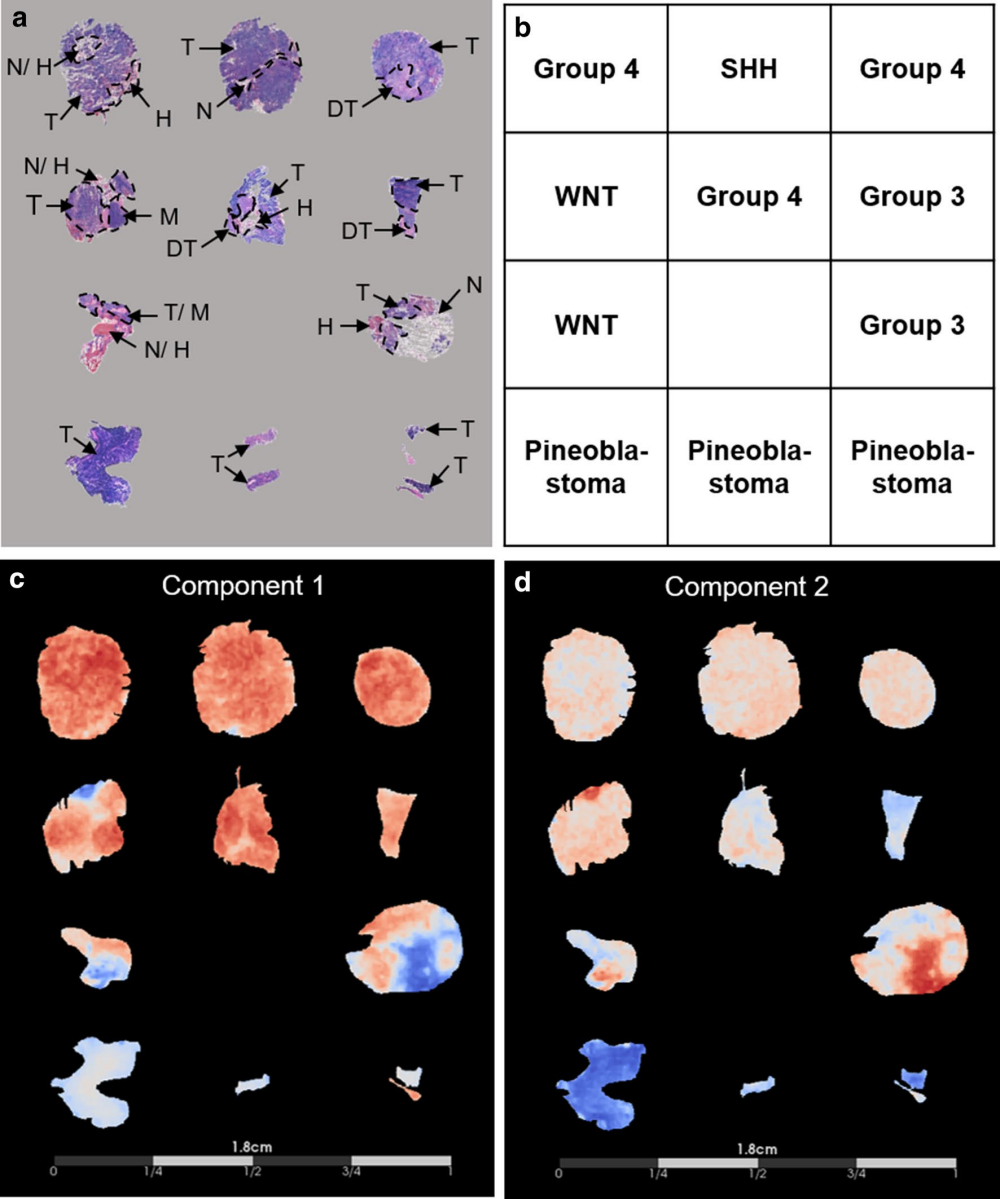
Histological characterization of medulloblastoma and pineoblastoma compared to principal component analysis of the mass spectrometry imaging dataset. **a** Tissue sections were hematoxylin and eosin stained and optical images at ×40 magnification were assessed to delineate histopathological features, indicated by the dashed lines and arrows, where: tumor (T), differentiated tumor (DT), necrosis (N), hemorrhage (H), and cerebral cortex molecular layer (M). **b** Layout of specimens. **c**, **d** Display of the PCA results using a cool-to-warm intensity heat map for which the gradient blue-white-red represents negative-to-positive degrees of variance for the separate components. Component 1 (**c**), representative of the greatest variance of the MSI dataset, distinguished medulloblastoma from pineoblastoma as indicated by the overall abundance of red and blue present in the medulloblastoma specimens compared to white present in the pineoblastoma specimens. Component 2 (**d**), representative of the second greatest variance of the MSI dataset, also distinguished medulloblastoma from pineoblastoma observed by the overall red-white present in the medulloblastoma specimens compared to the blue gradient present in pineoblastoma

We performed MALDI MSI on MB and PB tissue sections to determine if the spectral profiles of the two tumor types differed. Following pathology review of post-analysis H&E stained sections, we focused the MALDI MSI data analyses on areas of dense tumor so as to identify molecules that were preferentially detected in tumor tissue. In brief, the MB tissue sections displayed varying histopathological features including regions of small, blue tumor cells, differentiated tumor, necrosis, and hemorrhage, and the PB tissue sections displayed regions of dense tumor and tumor infiltrating into normal tissue (Fig. 1).

We performed PCA using a preprocessed peak list, thereby taking into consideration the underlying variance patterns within the MALDI MSI data that may not be obvious from analysis of single ion images. Component one of PCA, which represents the most discriminating features in the MALDI MSI data, reflected 44.89% of the discriminatory power of the model and separated MB from PB (Fig. 1). PCA component two, which represents the second greatest variance in the MALDI MSI data, reflected an additional 23.50% of the discriminatory power of the model and also separated MB from PB.

We performed ROC analysis on the peak list to find potential biomarkers for these two tumor types. We calculated AUC values for each *m*/z interval and the calculations ranged from 0.97 to 0.03, where an AUC of 1.0 indicated a perfect classifier for MB and an AUC of 0.0 indicated a perfect classifier for PB (Online Resource Table 1). Sixty-eight molecular ions had AUC values above 0.75 and were considered as potential classifiers of MB. Thirty ions had AUC values below 0.25 and were considered as potential classifiers of PB. The remaining 45 ions with AUC values falling between 0.75 and 0.25 were investigated as ions that are associated with cancer cells in general and that did not specifically discriminate MB and PB.

### Identification of ions

Multivariate analysis of the MALDI MSI data from MB and PB revealed differences between these two tumor types. We queried the entire peak list of potential classifiers against known biomolecules in the Lipid Maps database, seeking to identify each of these molecules. We considered ions with *m*/*z* intervals with AUC > 0.75 or AUC < 0.25 as potential biomarkers of MB and PB, respectively. We also characterized ions with AUC values between these thresholds (0.25–0.75). While such molecules do not discriminate MB and PB, they may still provide insights into the shared biology of these tumors and be potentially useful diagnostically, providing guidance when determining intraoperative surgical margins.

This query resulted in 22 peak assignments based on criteria outlined in the methodology section and categorized: 10 ions MB classifiers (Online Resource Table 2); two ions PB classifiers (Online Resource Table 3); 10 ions non-classifiers (Online Resource Table 4). Glycerophosphoglycerols comprised the largest number of assignments for MB classifiers, with five of 10 ions belonging to this class of phospholipids. Of those five glycerophosphoglycerol species, three were the top MB classifiers. The remaining five peak assignments for MB classifiers were identified as glycerophosphocholines (3/10) and sphingolipids (2/10). The two PB classifiers belong to the phospholipid main class of sphingolipids. Non-classifier ions that do not distinguish tumor types, but could distinguish tumor from normal tissue were identified as belonging to the phospholipid main classes of sphingolipids (4/10), glycerophosphoglycerols (3/10), glycerophosphocholines (2/10), and lysophosphatidic acids (1/10).

### Differential ion intensities informed by multivariate analyses of MALDI MSI

**Fig. 2:**
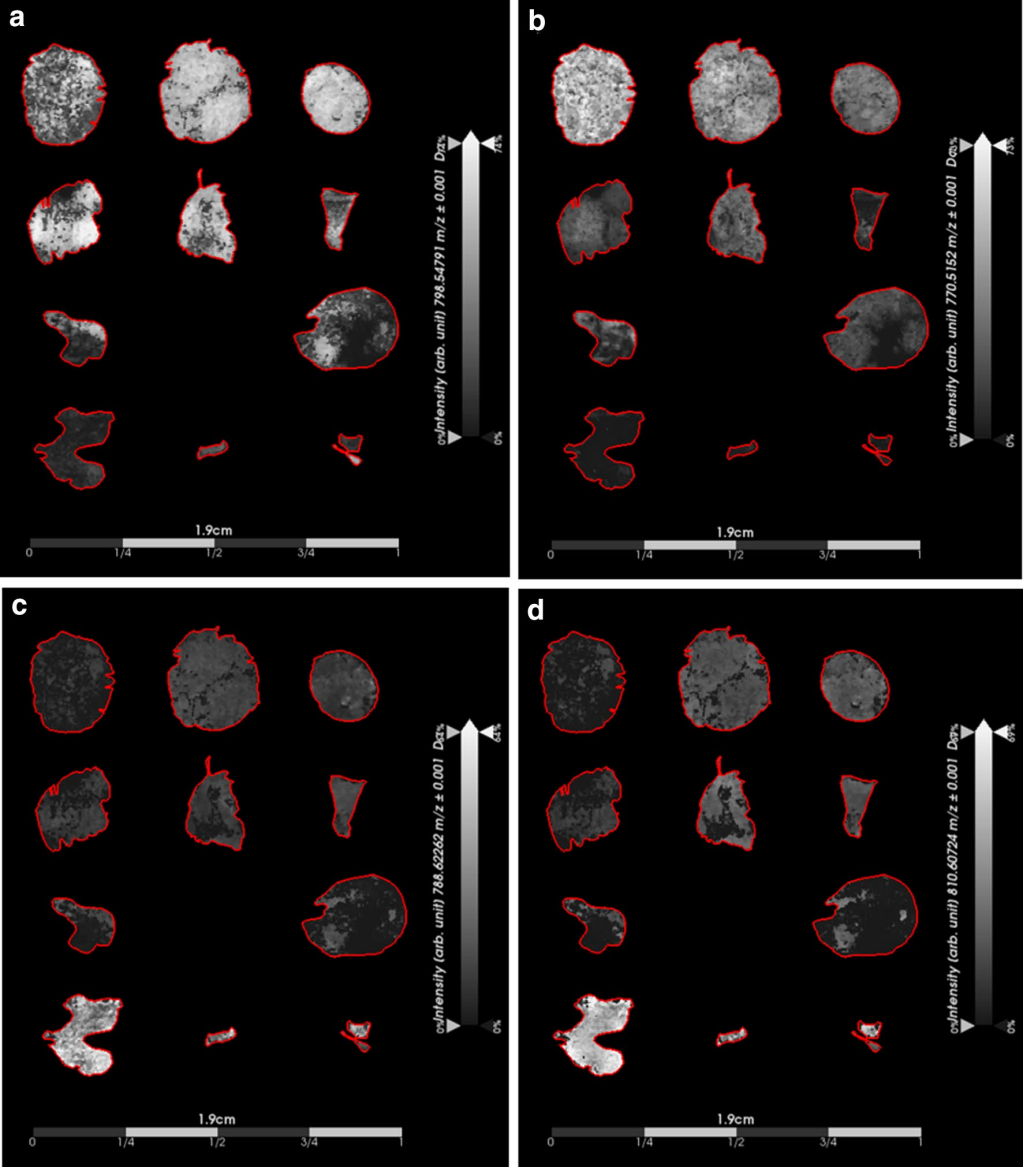
Single ion images generated from ions identified by principal component analysis (PCA) of the mass spectral imaging (MSI) dataset. Results demonstrate the capability of using potential biomarkers to discriminate between medulloblastoma and pineoblastoma tumor types. Important to note that the ion intensity scales were adjusted relative to each individual ion intensity. **a** Displays intensity of an ion detected at *m*/*z* 798.5479 that was detected with higher intensity in all subgroups of medulloblastoma when compared to pineoblastoma. **b** Displays the intensity of an additional ion (*m*/*z* 770.5152) that was detected with higher intensity in all subgroups of medulloblastoma when compared to pineoblastoma. **c** and **d** Displays intensities of two ions detected with higher intensity in pineoblastoma when compared to medulloblastoma with *m*/*z* of 788.6226 and *m*/*z* 810.6072, respectively

To facilitate discovery of additional ions to the two tumor types, we focused on ions that contributed to the variance highlighted by PCA, but that were not identified in the Lipid Maps database. We show single ion images, indicating two ions higher in MB (*m*/*z* 798.5479 and *m*/*z* 770.5152) and two ions higher in PB (*m*/*z* 788.6226 and *m*/*z* 810.6072) (Fig. 2). Although these ions were not identified in our database search, they may still be useful as potential biomarkers given that their AUC values fell within the specified thresholds of AUC > 0.75 (MB classifier) or AUC < 0.25 (PB classifier) and their correlation with histopathology evaluation.

**Fig. 3:**
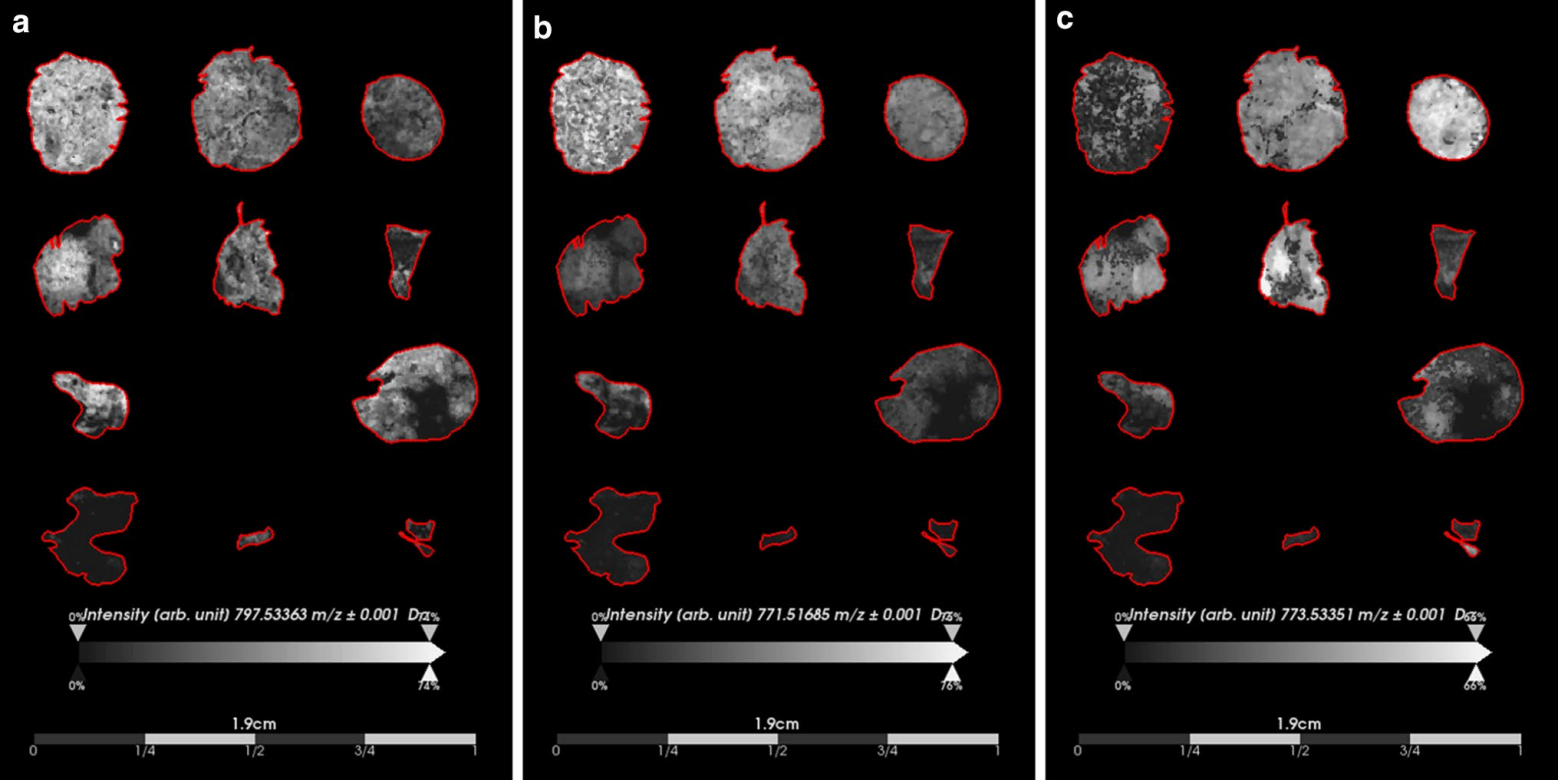
Distribution and intensity of top three ranked potential medulloblastoma classifiers based on receiver operating characteristics (ROC) analysis. **a** The highest ranked classifier for medulloblastoma, PG(38:5) (*m*/*z* 797.5336), displayed homogeneous distribution closely related to regions of dense tumor in all subgroups of medulloblastoma. **b** The second highest medulloblastoma classifier, PG(36:4) (*m*/*z* 771.5169), showed similar distribution to PG(38:5) as it was also present in regions of dense tumor in all medulloblastoma subgroups. **c** The third highest medulloblastoma classifier, PG(36:3) (*m*/*z* 773.5335), was also observed in all sections of medulloblastoma. The spatial distribution of these lipid species in medulloblastoma compared to pineoblastoma suggest their potential value as medulloblastoma biomarkers

**Fig. 4:**
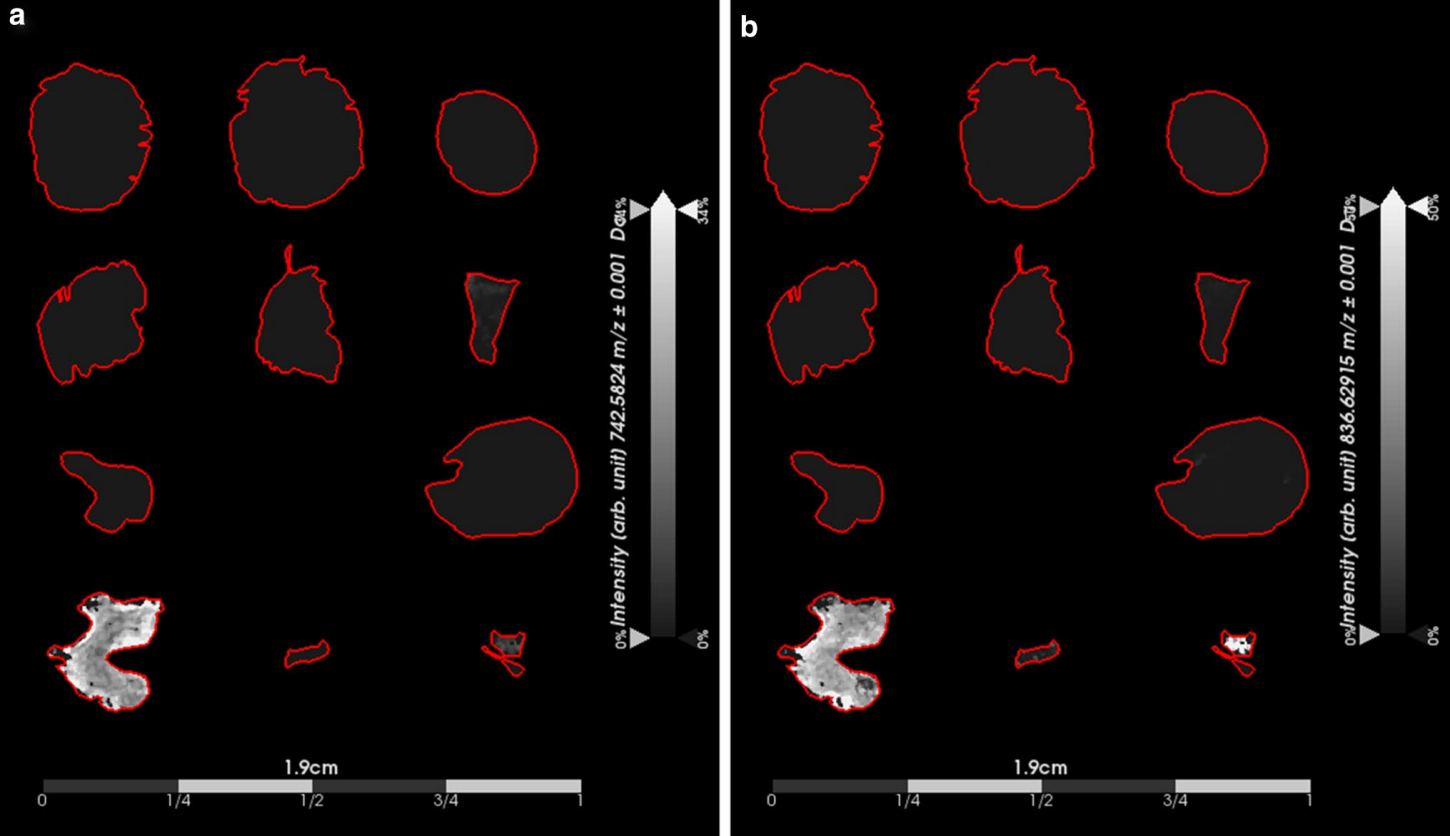
Distribution and intensity of potential pineoblastoma classifiers based on receiver operating characteristics (ROC) analysis. The two identified classifiers of pineoblastoma, HexCer(t36:2) (*m*/*z* 742.5824) **a** and CerP(d47:2) (*m*/*z* 836.6292) **b**, displayed similar spatial distribution and intensity present in only the pineoblastoma specimens

We next visualized the single ion images of the lipid species that we had identified as classifiers for MB and PB (Online Resources Tables 2 and 3, respectively) and assessed their spatial distribution and intensity. The highest ranking classifier for MB, a phosphoglycerol (PG) species, PG(38:5) (*m*/*z* 797.5336) with an AUC_max_ of 0.93, displayed an overall high signal intensity that was present homogeneously throughout dense tumor regions. Likewise, the second and third highest classifiers for MB, PG(36:4) ]*m*/*z* 771.5169 (AUC_max_ = 0.89)] and PG(36:3) [*m*/*z* 773.5335 (AUC_max_ = 0.86)], respectively, displayed similar single ion images with high signal intensities present homogeneously throughout the dense tumor regions (Fig. 3). None of these ions were detected in PB tumor tissue. The single ion images of the two sphingolipids that we had identified as PB classifiers, namely HexCer(t36:2) [*m*/*z* 742.5824 (AUC_max_ = 0.04)] and CerP(d47:2) [*m*/*z* 836.6292 (AUC_max_ = 0.06)], revealed high signal intensity that was present homogeneously throughout dense tumor regions of PB tissue sections. Neither ion was detected in MB tissue sections (Fig. 4).

**Fig. 5:**
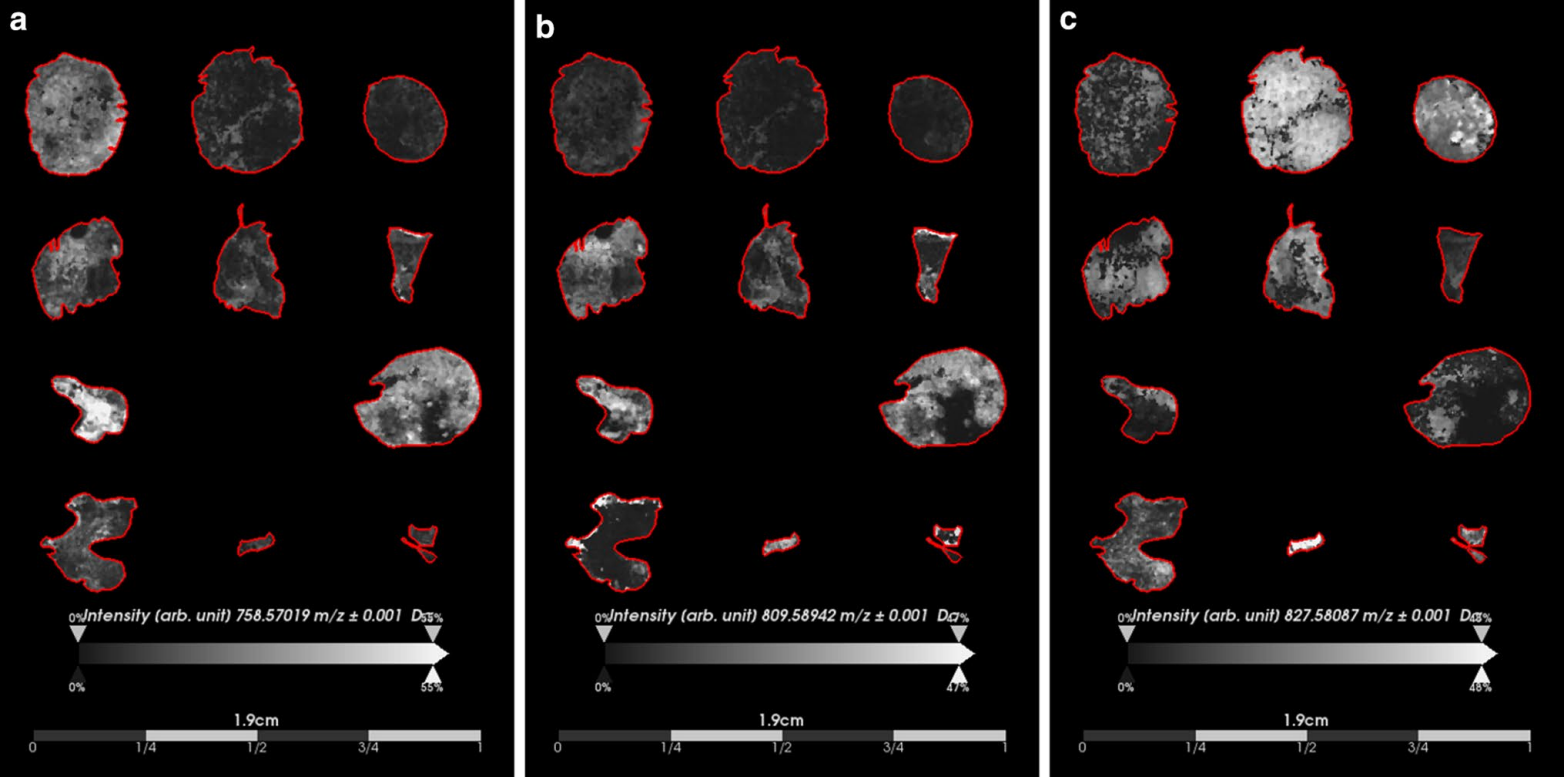
Non-classifier ions indicative of histopathology rather than tumor type. The ion images of lipid species not discriminative of tumor type were investigated to determine if their distribution could be of interest to underlying biological similarities between medulloblastoma and pineoblastoma. **a** PC(34:2) (*m*/*z* 758.5702) displayed varying intensity across medulloblastoma and pineoblastoma tissue sections that more closely resembled areas of necrosis and hemorrhage rather than viable tumor. **b** PG(37:0(OH)) (*m*/*z* 809.5894) also displayed distribution in the tissue sections not indicative of viable tumor regions, but more related to potential necrosis, or hemorrhage. **c** PG(40:4) (*m*/*z* 827.5809) was observed in both medulloblastoma and pineoblastoma specimens and its distribution was more closely related to regions of viable tumor

We explored the spatial distribution and intensity of the lipid species that we had characterized as non-classifiers by ROC analysis (Fig. 5). We selected ions with AUC_max_ approximately equivalent to 0.50 (best ranking non-classifiers) because they did not discriminate the tumor types and therefore might provide insights into common biological features that are shared amongst the two different tumor types. The varying spatial distribution and signal intensity of these ions were more reflective of histopathology features rather than tumor type. For instance, the phosphocholine (PC) species, PC(34:2) [*m*/*z* 758.5702 (AUC_max_ = 0.51)], displayed higher signal intensity in regions of the tissue sections that were not considered to be regions of viable tumor, but more closely aligning to regions of necrosis or hemorrhage (Fig. 5a). In addition, for PG(37:0(OH)) [*m*/*z* 809.5894 (AUC_max_ = 0.50)], we also observed higher signal intensity in regions of tissue that were not characterized as viable tumor, but the spatial distribution differed from that of PC(34:2) (*m*/*z* 758.5702) (Fig. 5b). On the other hand, PG(40:4) [*m*/*z* 827.5809 (AUC_max_ = 0.48)] displayed varying signal intensity with a spatial distribution throughout all of the tissue sections and its higher signal intensity more closely aligned to regions of viable tumor (Fig. 5c), thus representing a tumor marker for both MB and PB.

## Discussion

### Discrimination between medulloblastoma and pineoblastoma

While recent whole genome sequencing studies have allowed for the molecular classification of MB tumor types, histopathological review remains the first method for characterizing a gross tumor sample once obtained by biopsy or resection. Histopathology results typically guide diagnosis and the recommended course of treatment. Histologically, MB and PB appear nearly indistinguishable as embryonal tumors, but have very different treatment plans and expected outcomes. Misdiagnoses of these tumor types based on histopathology have occurred [5], highlighting the need for a more rapid and accurate characterization method to discriminate MB and PB. Mass spectrometry has been used to analyze human glioblastoma surgical samples to assist in decision making, particularly for classification of glioblastomas with unknown isocitrate dehydrogenase (*IDH)* status by quantitatively monitoring surgically resected tissue for the presence of the onco-metabolite, 2-hydroxyglutarate, produced by *IDH* mutant glioblastomas [28–30]. The results described herein demonstrate the capability of discriminating MB and PB tumor types by characterizing their lipid profile using MALDI MSI. Multivariate analyses of the MSI dataset demonstrated the greatest variance between the two tumor types, as PCA components one and two which both differentiated MB and PB.

While PCA is a powerful tool to visualize underlying trends in MSI data, it is performed post-acquisition on preprocessed MSI data and is not readily applicable to clinical applications requiring real-time feedback. Robust biomarkers capable of differentiating these two tumor types are needed to facilitate rapid and accurate diagnoses. Furthermore, biomarker identification for discriminating these two tumor types is, to the best of our knowledge, an overlooked area. To address this unmet need, we used ROC analysis to extract the relationship of any given ion as a potential classifier of either MB or PB. Querying these ions against known lipid species using Lipid Maps database facilitated the discovery of potential diagnostic biomarkers. Ten lipid species were identified as potential classifiers of MB and two lipid species were identified as potential classifiers of PB. The classifiers identified in this study suggest particular classes of lipid species may be useful for distinguishing these two tumor types, specifically glycerophosphoglycerols and glycerophosphocholines for identifying MB and sphingolipids for PB.

### Lipid aberrations facilitate biomarker identification

Important roles of lipids in cancer development and pathogenesis, including their role in specific cell signaling pathways, such as SHH and WNT, have recently been identified [31–34]. Oxysterols have been implicated in the survival of SHH MB subgroup [35] and aerobic glycolysis, or the Warburg effect, has been observed in the WNT MB subgroup [36–39]. While our results did not identify cholesterol or other sterol molecules, which may be related to the inherent difficulty of analyzing these species by MALDI [40], PG species were the most abundant identified phospholipids in MB, followed by PC species. These results align with a recent study of metabolomics in embryonal tumors which measured PC as the second highest metabolite in MB, compared to retinoblastoma and neuroblastoma, when metabolism was analyzed by high resolution magnetic resonance spectroscopy (^1^H-MRS) [41]. Interestingly, lactate was the metabolite with the highest concentration in the three aforementioned tumor types [41] suggesting these tumors may be switching their metabolic preference to promote the production of biomass (i.e. result of the Warburg effect). To the best of our knowledge, glycerophosphoglycerols have not been implicated in MB, but have been positively associated with cancer cell migration [42]. Molecular and biochemical characterization of PB has been investigated to a lesser extent than MB. Our results indicate sphingolipids may be important as distinguishing biomarkers of PB. Sphingolipids have shown to be related to critical roles surrounding oncogenesis, such as cell growth, proliferation, and death [43, 44] and may be relevant to what the underlying molecular aberrations are in PB.

### Mass spectrometry can distinguish medulloblastoma and pineoblastoma

Our results demonstrate a rapid and information-rich approach, mass spectrometry imaging, to distinguish these two tumor types based on differences in lipid profiles. This approach has identified interesting differences in lipid profiles, which could potentially be interesting from a biological and future drug therapeutic standpoint. However, one limitation of this work is the lack of age-matched healthy control brain tissue. Thus, whether the differences observed here are due to tumor type or the distinct anatomical location of samples analyzed will need further investigation.

MSI is particularly advantageous for spatially resolving molecules within samples allowing for label free molecular images that can be compared with more traditional histopathology and immunohistochemistry assessment for initial validation. While we present the application of MALDI MSI of frozen tissue samples, it could be possible to translate this approach to ‘real-time’ MS analyses to assist in distinguishing MB from PB with the recent development of a more rapid MALDI-TOF instrument [45]. The near real-time capability of MS to provide clinically relevant results (e.g. tumor versus healthy tissue and MB versus PB) would reduce the time between biopsy and diagnosis, allowing patients to be placed on relevant clinical trials sooner. Even though treatment may not be initiated sooner, knowing the subgroup of the MB for example, may enable the patient to be screened for a relevant clinical trial.

## Conclusion

In summary, these results demonstrate a proof-of-concept for the feasibility of using a MS based approach to distinguish MB and PB, two histopathologically similar tumor types. Glycerophosphoglycerols were identified as the top MB classifiers while sphingolipids were identified as the top PB classifiers. The frozen patient samples used in this study is quite rare, limiting the conclusions that can be drawn. Future studies are needed to further validate our findings across a larger number of samples. In addition, more effort is warranted to explore the distinct metabolomic signature profiles of MB and PB and whether such profiles can provide the framework for establishing rapid biochemical based diagnostic, prognostic, and predictive signatures.

## Electronic supplementary material

Below is the link to the electronic supplementary material.

Supplementary material 1 (DOCX 19 KB)

Supplementary material 2 (DOCX 16 KB)

Supplementary material 3 (DOCX 15 KB)

Supplementary material 4 (DOCX 16 KB)

## References

[1] Ostrom QT, Gittleman H, Xu J (2016). CBTRUS statistical report: primary brain and other central nervous system tumors diagnosed in the United States in 2009–2013. Neuro Oncol.

[2] Bailey P, Cushing H (1925). Medulloblastoma cerebelli: a common type of midcerebellar glioma of childhood. Arch Neurol Psychiatry.

[3] Louis D, Ohgaki H, Wiestler O et al (2016) WHO classification of tumours of the central nervous system. In: Louis DN, Ohgaki H, Wiestler OD, Cavenee WK (eds) WHO classification of tumours of the central nervous system, 4th edn. International Agency For Research On Cancer, Lyon Cedex 08, pp 184–200

[4] Northcott PA, Buchhalter I, Morrissy AS (2017). The whole-genome landscape of medulloblastoma subtypes. Nature.

[5] Raleigh DR, Solomon DA, Lloyd SA (2017). Histopathologic review of pineal parenchymal tumors identifies novel morphologic subtypes and prognostic factors for outcome. Neuro-Oncology.

[6] Louis DN, Ohgaki H, Wiestler OD et al (2016) WHO classification of tumours of the central nervous system. In: Louis DN, Ohgaki H, Wiestler OD, Cavenee WK (eds) WHO classification of tumours of the central nervous system, 4th edn. International Agency For Research On Cancer, Lyon Cedex 08, pp 176–179

[7] Sengupta S, Pomeranz Krummel D, Pomeroy S (2017). The evolution of medulloblastoma therapy to personalized medicine. F1000Research.

[8] Fontana EJ, Garvin J, Feldstein N, Anderson RCE (2011). Pediatric considerations for pineal tumor management. Neurosurg Clin N Am.

[9] Misawa K, Mochizuki D, Imai A (2017). Epigenetic silencing of SALL3 is an independent predictor of poor survival in head and neck cancer. Clin Epigenetics.

[10] Vallius T, Hynninen J, Auranen A (2017). Postoperative human epididymis protein 4 predicts primary therapy outcome in advanced epithelial ovarian cancer. Tumor Biol.

[11] Currie E, Schulze A, Zechner R (2013). Cellular fatty acid metabolism and cancer. Cell Metab.

[12] Luo X, Cheng C, Tan Z (2017). Emerging roles of lipid metabolism in cancer metastasis. Mol Cancer.

[13] Ray U, Roy SS (2017). Aberrant lipid metabolism in cancer cells—the role of oncolipid-activated signaling. FEBS J.

[14] Fernandes Messias MC, Mecatti GC, Figueiredo Angolini CF (2018). Plasma lipidomic signature of rectal adenocarcinoma reveals potential biomarkers. Front Oncol.

[15] Lee MY, Yeon A, Shahid M (2018). Reprogrammed lipid metabolism in bladder cancer with cisplatin resistance. Oncotarget.

[16] Jiang N, Zhang G, Pan L (2017). Potential plasma lipid biomarkers in early-stage breast cancer. Biotechnol Lett.

[17] Stephenson DJ, Alexis Hoeferlin L, Chalfant Richmond CE (2017). Lipidomics in translational research and the clinical significance of lipid-based biomarkers. Transl Res.

[18] Xiao Y, Chen Y, Kennedy a W (2000). Evaluation of plasma lysophospholipids for diagnostic significance using electrospray ionization mass spectrometry (ESI-MS) analyses. Ann N Y Acad Sci.

[19] Li J, Ren S, Piao H (2016). Integration of lipidomics and transcriptomics unravels aberrant lipid metabolism and defines cholesteryl oleate as potential biomarker of prostate cancer. Sci Rep.

[20] Hall Z, Ament Z, Wilson CH (2016). MYC expression drives aberrant lipid metabolism in lung cancer. Cancer Res.

[21] Liu Y, Chen Y, Momin A (2010). Elevation of sulfatides in ovarian cancer: an integrated transcriptomic and lipidomic analysis including tissue-imaging mass spectrometry. Mol Cancer.

[22] Goto-Inoue N, Hayasaka T, Zaima N, Setou M (2011). Imaging mass spectrometry for lipidomics. Biochim Biophys Acta.

[23] Dilillo M, Ait-Belkacem R, Esteve C (2017). Ultra-high mass resolution MALDI imaging mass spectrometry of proteins and metabolites in a mouse model of glioblastoma. Sci Rep.

[24] Patterson NH, Alabdulkarim B, Lazaris A (2016). Assessment of pathological response to therapy using lipid mass spectrometry imaging. Sci Rep.

[25] McDonnell LA, Angel PM, Lou S, Drake RR (2017). Mass spectrometry imaging in cancer research: future perspectives. Adv Cancer Res.

[26] Pugh TJ, Weeraratne SD, Archer TC (2012). Medulloblastoma exome sequencing uncovers subtype-specific somatic mutations. Nature.

[27] Sud M, Fahy E, Cotter D (2007). LMSD: LIPID MAPS structure database. Nucleic Acids Res.

[28] Eberlin LS, Norton I, Dill AL (2012). Classifying human brain tumors by lipid imaging with mass spectrometry. Cancer Res.

[29] Calligaris D, Norton I, Feldman DR (2013). Mass spectrometry imaging as a tool for surgical decision-making. J Mass Spectrom.

[30] Santagata S, Eberlin LS, Norton I (2014). Intraoperative mass spectrometry mapping of an onco-metabolite to guide brain tumor surgery. Proc Natl Acad Sci USA.

[31] Blassberg R, Jacob J (2017). Lipid metabolism fattens up hedgehog signaling. BMC Biol.

[32] Long J, Tokhunts R, Old WM (2015). Identification of a family of fatty-acid-speciated sonic hedgehog proteins, whose members display differential biological properties. Cell Rep.

[33] Sethi JK, Vidal-Puig A (2010). Wnt signalling and the control of cellular metabolism. Biochem J.

[34] Beloribi-Djefaflia S, Vasseur S, Guillaumond F (2016). Lipid metabolic reprogramming in cancer cells. Oncogenesis.

[35] Corcoran RB, Scott MP (2006). Oxysterols stimulate Sonic hedgehog signal transduction and proliferation of medulloblastoma cells. Proc Natl Acad Sci USA.

[36] Lee SY, Jeon HM, Ju MK (2012). Wnt/snail signaling regulates cytochrome c oxidase and glucose metabolism. Cancer Res.

[37] Sherwood V, Chaurasiya SK, Ekström EJ (2014). WNT5A-mediated ß-catenin-independent signalling is a novel regulator of cancer cell metabolism. Carcinogenesis.

[38] Yang L, Perez AA, Fujie S (2014). Wnt modulates MCL1 to control cell survival in triple negative breast cancer. BMC Cancer.

[39] Pate KT, Stringari C, Sprowl-Tanio S (2014). Wnt signaling directs a metabolic program of glycolysis and angiogenesis in colon cancer. EMBO J.

[40] Xu L, Kliman M, Forsythe JG (2015). Profiling and imaging ion mobility-mass spectrometry analysis of cholesterol and 7-dehydrocholesterol in cells via sputtered silver MALDI. J Am Soc Mass Spectrom.

[41] Kohe SE, Bennett CD, Gill SK (2018). Metabolic profiling of the three neural derived embryonal pediatric tumors retinoblastoma, neuroblastoma and medulloblastoma, identifies distinct metabolic profiles. Oncotarget.

[42] Hutschenreuther A, Birkenmeier G, Bigl M (2013). Glycerophosphoglycerol, beta-alanine, and pantothenic acid as metabolic companions of glycolytic activity and cell migration in breast cancer cell lines. Metabolites.

[43] Ponnusamy S, Meyers-Needham M, Senkal CE (2010). Sphingolipids and cancer: ceramide and sphingosine-1-phosphate in the regulation of cell death and drug resistance. Futur Oncol.

[44] Ogretmen B (2017). Sphingolipid metabolism in cancer signalling and therapy. Nat Rev Cancer.

[45] Ogrinc Potočnik N, Porta T, Becker M (2015). Use of advantageous, volatile matrices enabled by next-generation high-speed matrix-assisted laser desorption/ionization time-of-flight imaging employing a scanning laser beam. Rapid Commun Mass Spectrom.

