# Supplementary material for: Rapid discrimination of pediatric brain tumors by mass spectrometry imaging
Source: J Neurooncol. 2018 Aug 20;140(2):269–79. doi: 10.1007/s11060-018-2978-2 (PMC6244779; doi:10.1007/s11060-018-2978-2)
Supplement: Supplementary file 2 — Supplementary material 2 (DOCX 16 KB) [file 11060_2018_2978_MOESM2_ESM.docx]

**Rapid discrimination of medulloblastoma and pineoblastoma by mass spectrometry imaging**

**Journal of Neuro-Oncology**

Amanda R. Clark, BS, David Calligaris, PhD, Michael S. Regan, BS, Daniel Pomeranz Krummel, PhD, Jeffrey N. Agar, PhD, Laura Kallay, PhD, Tobey MacDonald, MD, Matthew Schniederjan, MD, Sandro Santagata, MD, PhD, Scott L. Pomeroy, MD, PhD, *Nathalie Y. R. Agar, PhD, *Soma Sengupta, MBBS, PhD, MRCP

*NYRA and SS are co-corresponding authors.

Co-corresponding author affiliation and e-mail address:

**Nathalie Y. R. Agar**

Department of Neurosurgery

Brigham and Women’s Hospital

60 Fenwood Road, 8016-J

Boston, MA 02115

Email: [Nathalie_Agar@dfci.harvard.edu](mailto:Nathalie_Agar@dfci.harvard.edu)

**Soma Sengupta**

Winship Cancer Institute

Emory University Hospital

1365C Clifton Road, Suite C5086

Atlanta, GA 30322

Email: soma.sengupta@emory.edu

**Online Resource 2. Potential classifiers of medulloblastoma.** Tentative peak assignments and main class categories for lipid species discriminative of MB compared to PB based on Lipid Maps database search.

| AUC_maximum_ | *m/z*_measured_ | [M+X]^+^ (X = H^+^, Na^+^, or K^+^) | *m/z*_calculated_ | Δppm | Tentative Peak Assignment | Main Class |
| --- | --- | --- | --- | --- | --- | --- |
| 0.93 | 797.5336 | [M+H]^+^ | 797.5327 | 1.6 | PG(38:5) | Glycerophosphoglycerol |
| 0.89 | 771.5169 | [M+H]^+^ | 771.5171 | 0.1 | PG(36:4) | Glycerophosphoglycerol |
| 0.86 | 773.5335 | [M+H]^+^ | 773.5327 | 1.7 | PG(36:3) | Glycerophosphoglycerol |
| 0.85 | 770.5710 | [M+H]^+^ | 770.5695 | 1.9 | PC(35:3) | Glycerophosphocholine |
| 0.85 | 754.5380 | [M+H]^+^ | 754.5382 | 0.3 | PC(34:4) | Glycerophosphocholine |
| 0.84 | 823.5500 | [M+H]^+^ | 823.5484 | 1.9 | PG(40:6) | Glycerophosphoglycerol |
| 0.80 | 798.5479 | [M+H]^+^ | 798.5491 | 1.4 | PI-Cer(t34:0) | Sphingolipid |
| 0.80 | 780.5545 | [M+H]^+^ | 780.5538 | 1.5 | PC(36:5) | Glycerophosphocholine |
| 0.78 | 844.5319 | [M+Na]^+^ | 844.5310 | 1.2 | PI-Cer(t36:2) | Sphingolipid |
| 0.78 | 781.5589 | [M+H]^+^ | 781.5590 | 0.0 | PG(35:0(OH)) | Glycerophosphoglycerol |
